# Purification and Characterization of Recombinant Expressed Apple Allergen Mal d 1

**DOI:** 10.3390/mps4010003

**Published:** 2020-12-27

**Authors:** Julia A. H. Kaeswurm, Bettina Nestl, Sven M. Richter, Max Emperle, Maria Buchweitz

**Affiliations:** 1 Department of Food Chemistry, Institute of Biochemistry and Technical Biochemistry, University of Stuttgart, 70569 Stuttgart, Germany; julia.kaeswurm@lc.uni-stuttgart.de; 2 Department of Technical Biochemistry, Institute of Biochemistry and Technical Biochemistry, University of Stuttgart, 70569 Stuttgart, Germany; bettina.nestl@ibtb.uni-stuttgart.de (B.N.); sven.richter@ibtb.uni-stuttgart.de (S.M.R.); max.emperle@ibtb.uni-stuttgart.de (M.E.)

**Keywords:** apple allergy, Mal d 1, recombinant expression in *E. coli*, purification, high-resolution anion-exchange column, CD spectra, tryptic in gel digestion, peptide identification, stability, dimer formation

## Abstract

Mal d 1 is the primary apple allergen in northern Europe. To explain the differences in the allergenicity of apple varieties, it is essential to study its properties and interaction with other phytochemicals, which might modulate the allergenic potential. Therefore, an optimized production route followed by an unsophisticated purification step for Mal d 1 and respective mutants is desired to produce sufficient amounts. We describe a procedure for the transformation of the plasmid in competent *E. coli* cells, protein expression and rapid one-step purification. r-Mal d 1 with and without a polyhistidine-tag are purified by immobilized metal ion affinity chromatography (IMAC) and fast-protein liquid chromatography (FPLC) using a high-resolution anion-exchange column, respectively. Purity is estimated by SDS-PAGE using an image-processing program (Fiji). For both mutants an appropriate yield of r-Mal d 1 with purity higher than 85% is achieved. The allergen is characterized after tryptic in gel digestion by peptide analyses using HPLC-MS/MS. Secondary structure elements are calculated based on CD-spectroscopy and the negligible impact of the polyhistidine-tag on the folding is confirmed. The formation of dimers is proved by mass spectrometry and reduction by DTT prior to SDS-PAGE. Furthermore, the impact of the freeze and thawing process, freeze drying and storage on dimer formation is investigated.

## 1. Introduction

The allergen Mal d 1 is the primary apple allergen in northern Europe [1]. Due to the great structural similarity between the allergen Bet v 1 in birch and Mal d 1 in apple, the immune system can only distinguish between the two proteins with difficulty [2,3,4]. Therefore, about 50−70% of people affected by birch pollen allergy develop a cross-allergy to apple (*Malus domestica*) during their lifetime [1,5,6]. Although the 17.5 kDa large allergen Mal d 1 is heat-labile and proteases break it down during gastric digestion, [7] the symptoms during consumption, such as breathing difficulties, itching and burning of the mucous membranes of the mouth or tongue (Oral Allergy Syndrome) mean that an estimated 2 million affected people in Germany alone generally avoid eating fresh apples [1,5].

It is striking that the allergenic potential, which up to now has only been examined diagnostically on the effect level after oral provocation, skin prick tests or immunochemical testing with allergy sera, clearly differs between different apple varieties [8,9]. However, these differences could not be sufficiently correlated with the variety- or cultivation-related differences in the Mal d 1 content so that the absolute content of Mal d 1 cannot be the sole cause of an apple’s allergenic potential [10,11,12]. An interaction of Mal d 1 with other phytochemicals reducing the allergenic potential has been postulated [13,14,15], but the structural requirements for the phytochemicals, as well as the interaction mechanisms, are still unknown. To investigate interaction strength, kinetics, and desired reaction conditions for different phytochemicals, purified Mal d 1 is a prerequisite. Purification by immobilized metal ion affinity chromatography (IMAC) for proteins exhibiting a C-terminal His-Tag might be a quick procedure to obtain high purities. Nevertheless, in lots of experimental settings, this additional feature is perturbing. Therefore, a process to purify the protein without a His Tag is required as well. Commonly time-consuming multi-step purification methods, including FPLC (fast protein liquid chromatography) based on a combination of different separation principles like ion exchange (IEC), hydrophobic interaction and size exclusion chromatography, is used [16,17,18].

In contrast, in this manuscript the development of a rapid method to produce and purify sufficient amounts of r-Mal d 1 with and without a polyhistidine-tag in high purity for further experiments is described.

## 2. Experimental Design

**Figure 1:**
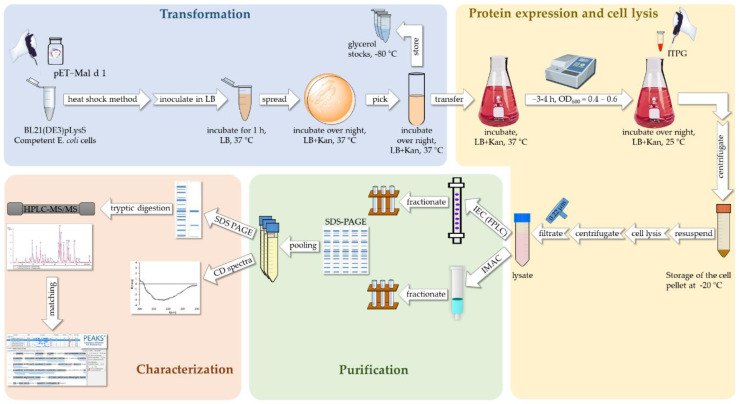
Purification and characterization workflow including plasmid transformation, r-Mal d 1 expression, purification, and characterization. Kan, Kanamycin; OD, optical density; ICE, ion exchange chromatography; IMAC, immobilized metal ion affinity chromatography; CD, circular dichroism.

The production protocol for r-Mal d 1 described here is divided into three parts, of which one is a protein purification step (Figure 1). After plasmid transformation in *Escherichia coli* (*E. coli*), the protein is expressed in the host cells, and the harvested cells are lysed by pressure. For the purification of the clear cell lysate containing r-Mal d 1 with a C-terminal polyhistidine-tag (r-Mal d 1-His), a HisTrap™ FF column is employed, performing IMAC. To purify the clear cell lysate with r-Mal d 1, a chromatographic system, the ÄKTA purifier FPLC system, and a high-resolution anion exchange column the TOYOPEARL^TM^ GigaCap Q-650M (IEC) are essential to perform this purification. However, no additional steps are required to obtain pure r-Mal d 1 after this step.

### 2.1. Materials

- Plasmid DNA in a pET-28b(+) vector with resistance against kanamycin, cloning sites are NcoI (CCATGG) and XhoI (CTCGAG), insert size is 485 bp (Mal d 1), 488 bp (Mal d 1–His), (BioCat GmbH, Heidelberg, Germany, Lot: MF72741) The amino acid sequence for Mal d 1 is according to the protein Q9SYW3 found in UniProt database, and the gene codon was optimized for expression in *E. coli*. For the polyhistidine variant, some valine-alanine interchanges were performed;
- *Escherichia coli* BL21 star (DE3)pLysS (ThermoFisher Scientific, Waltham, MA, USA);
- Potassium chloride (Sigma-Aldrich, Taufkirchen, Germany)
- Magnesium chloride hexahydrate (Sigma-Aldrich, Taufkirchen, Germany);
- Sodium chloride (Sigma-Aldrich, Taufkirchen, Germany)
- Tris(2-carboxyethyl)phosphine hydrochloride (TCEP) (Sigma-Aldrich, Taufkirchen, Germany);
- Imidazole (Sigma-Aldrich, Taufkirchen, Germany);
- Glycerol (Sigma-Aldrich, Taufkirchen, Germany);
- Tryptone (Fisher Scientific, Waltham, MA, USA);
- Yeast Extract (Fisher Scientific, Waltham, MA, USA);
- Lysogeny broth (LB) Lennox agar, granulated (Fisher Scientific, Waltham, MA, USA);
- Kanamycin (VWR International, Radnor, PA, USA);
- Isopropyl-d-1-thiogalactopyranoside (IPTG) (Fisher Scientific, Waltham, MA, USA);
- Tris hydrochloride (Tris-HCl) (Fisher Scientific, Waltham, MA, USA);
- Urea (Invitrogen, Carlsbad, CA, USA);
- Page Ruler Prestained Protein Ladder (Serva Dual Color Protein Standard III, Heidelberg, Germany);
- SERVAGel^TM^ TG PRiME^TM^ 4–20%, 15 sample wells (Serva, Heidelberg, Germany, Lot V200051).

### 2.2. Equipment

- Autoclave Getinge sterilizer (Getinge AB, Göteborg, Sweden)
- Emulsiflex-C5 (Avestin, Ottawa, ON, Canada);
- ÄKTA purifier 10 (GE Healthcare Biosciences AB, Uppsala, Sweden);
- HisTrap™ FF, Ni Sepharose™ 6.5 mL (GE Healthcare, Buckinghamshire, UK);
- TOYOPEARL^TM^ SuperQ-650S, strong anion exchange chromatography resin, SkillPak^TM^ 5 mL columns, particle size distribution 20–50 μm (Tosoh Bioscience GmbH, Griesheim, Germany);
- TOYOPEARL^TM^ GigaCap Q-650M, strong anion exchange chromatography resin, SkillPak^TM^ 5 mL columns, particle size distribution and 50–100 μm (Tosoh Bioscience GmbH, Griesheim, Germany);
- Non-sterile polyethersulfon syringe filter, 0.2 µm (VWR, Radnor, PA, USA);
- Sterile polyethersulfon syringe filter, 0.2 µm (VWR, Radnor, PA, USA);
- Incubation shaker New Brunswick™ Excella^®^ E24/E24R (Eppendorf, Hamburg, Germany);
- Viva spin Amicon Ultra-15 centrifugal filters, 10K cutoff (Millipore, Burlington, MA, USA);
- Labconco Freezone 12 Liter Console Freeze Dry System (Freeze-drying) (Labconco, Kansas City, MO, USA);
- Centrifuge Beckman Avanti J-26S XP (Beckman Coulter, Brea, CA, USA);
- Visible Spectrophotometer (JASCO, Easton, MD, USA; Cat. no.: V-730) for optical density (OD) determination;
- BMG reader SPECTROstar Nano equipped with an LVis Plate (680-101) and software version 5.50, and MARS version 3.33 for data analyses respectively (BMG Labtech; Ortenberg, Germany);
- GenPure UV/UF CAD plus Ultrapure Water Purification System (Thermo Fisher Scientific, Waltham, MA, USA);
- Thermomixer comfort (Eppendorf AG, Hamburg, Germany);
- SDS PAGE chamber Peqlab PerfectBlue Twin S, model 45–1010 (VWR, Radnor, PA, USA);
- SDS Gels SERVAGel TG PRiME, 4−12% (Serva, Heidelberg, Germany);
- J-815 circular dichroism spectrophotometer (Jasco Corporation, Tokyo, Japan) fitted with a PTC-423S Peltier-type single-position cell holder (Jasco; Essex, UK);
- Quartz cuvette with 0.1 mm path length (HellmaAnalytik, Mülheim, Germany);
- YMC-Triart C18 column (Cat. No. TA12S03-15; pore 12 nm, particle 3 μm, 150 mm length × 0.3 mm internal diameter);
- M3 MicroLC system (Sciex, Darmstadt, Germany);
- TripleTOF™ 6600 (Sciex, Darmstadt, Germany) coupled to the LC system by a DuoSpray™ Ion Source (Sciex).

## 3. Procedure

### 3.1. Preparation of Solutions 

Use ultrapure water to prepare all the solutions in the protocol. Make liquid Lennox Broth by dissolving 10 g tryptone, 5 g NaCl, and 5 g yeast extract in 1 L water. Autoclave the liquid LB and let it cool to room temperature. To prepare solid LB agar plates, add 15 g of agar per 1 L. For bacteria culture autoclave the broth and cool before adding 25 µg/mL sterile-filtered kanamycin and the transformed *E. coli*. For cell lysis steps and purification, prepare the following buffers:

- 100 mM Tris Cl pH 7.5, containing 100 mM NaCl;
- 100 mM Tris Cl pH 7.5, containing 100 mM NaCl and 10 mM imidazole;
- 100 mM Tris Cl pH 7.5, containing 100 mM NaCl and 100 mM imidazole;
- 20 mM Tris Cl, pH 7.5 containing 1 M NaCl;
- 100 mM phosphate buffer pH 7.5;
- 1 M urea buffer, supplemented with 166.7 mM thiourea and 8 mM Tris Cl, pH 8.

### 3.2. Expression of r-Mal d 1 and r-Mal d 1-His 

#### 3.2.1. Transformation of pET-Mal d 1 Plasmid into Competent *E. coli* Cells

- The transformation is performed using the Heat Shock Method [19]
- To transform the plasmid into *E*. *coli,* thaw the frozen cells of competent BL21(DE3)pLysS (50 µL) on ice, add 2 µL of the plasmid (0.1 ng/mL) to the cells and mix gently.
- Incubate the mixture on ice for 30 min and then heat the tubes for 45 s in a thermo shaker at 42 °C and 180 rpm to enable the transformation of plasmid into the cells.
- After heating, place the tubes on ice for 2 min, inoculate the transformed bacteria into 500 µL of liquid LB media without any antibiotics and incubate the cells for 1 h at 37 °C and 180 rpm.
- Centrifuge the cell suspension, dispose the supernatant, resuspend the transformed cells in 200 µL LB media and spread the cells on solid LB agar plates containing 25 µg/mL kanamycin, and keep the plates overnight in the incubator at 37 °C.

#### 3.2.2. Expression of Mal d 1 Protein by the Transformed *E. coli*

- Pick a colony, inoculate into 5 mL liquid LB and incubate at 37 °C and 180 rpm overnight.
- Add an aliquot of 600 µL of this starting culture to a 2 L no baffled flask containing 600 mL LB medium supplemented with 25 μg/mL kanamycin. Incubate flasks for each mutant at 37 °C and 180 rpm until an optical density (OD600) of 0.4–0.6 is reached.
- Start the overexpression of r-Mal d 1 by adding 0.5 mM isopropyl β-d-1-thiogalactopyranoside (IPTG) and incubate overnight at 25 °C and 180 rpm.
- After cell harvest by centrifugation at 4 °C (8980× *g*, 20 min), the washed cell pellets can be stored at −20 °C until further use.

#### 3.2.3. Cell Lyses

- Resolve the cell pellet (200 mg/mL) in 20 mM Tris buffer pH 7.5, disrupt physically three times with an EmulsiFlex (>600 bar) in a cooling chamber and centrifuge (30 min, 40,000× *g*, 4 °C).
- Filter the supernatant (filter, 0.2 µm) and use the lysate immediately for purification by immobilized metal ion affinity chromatography (IMAC) on a HisTrap™ FF (r-Mal d 1–His) or by ion exchange chromatography (IEC) on a TOYOPEARLTM SkillPakTM column (r-Mal d 1).

### 3.3. Purification of r-Mal d 1 

#### 3.3.1. Purification of r-Mal d 1–His by Immobilized Metal ion Affinity Chromatography (IMAC)

- Add one column volume (CV, 5 mL) lysate on a Tris buffer conditioned HisTrap™ FF column and wash with three CVs of Tris buffer (100 mM Tris∙Cl buffer pH 7.5 containing 100 mM NaCl).
- Eluate with three CVs of Tris buffer containing 10 mM imidazole, followed by five CVs Tris buffer containing 100 mM imidazole and collect fractions corresponding to the column volume.
- Check the purity by SDS-PAGE gel analysis and combine the respective fractions with a r-Mal d 1 purity higher than 85%.

#### 3.3.2. Purification of r-Mal d 1 by Ion-Exchange Chromatography (IEC)

- Add 5 mL lysate (corresponds to 1 g cell pellet) on an equilibrated TOYOPEARLTM SuperQ-650S or GigaCap Q-650M SkillPakTM and eluate with a flow of 2 mL/min with 1M NaCl in 20 mM Tris buffer, pH 7.5 (eluent B) using the gradients 4 or 5 (defined in Table 1; run 5, dilute 2.5 mL lysate with 2.5 mL 20 mM Tris buffer to check for overloading in run 1–4).
- Collect the fractions according the UV absorption, check purity by SDS-PAGE gel analysis and combine all fractions with a r-Mal d 1 purity higher than 85%, exchange buffer if required and store in aliquots at −20 °C.

**Figure 2:**
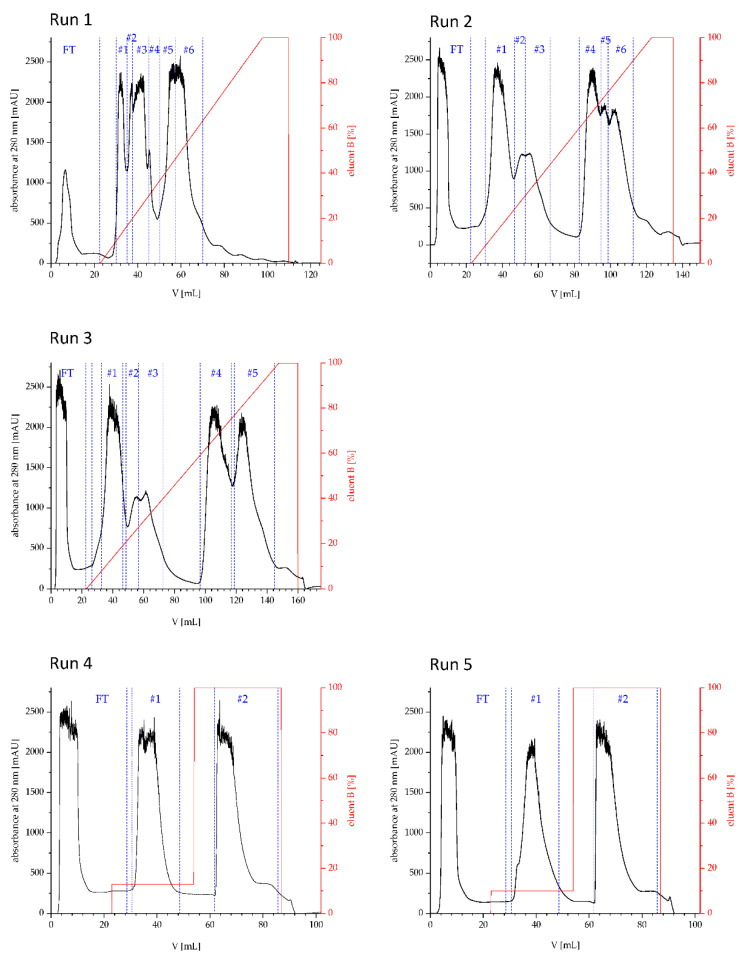
UV-chromatograms of run 1 to 5, including % B eluent (1 M NaCl in 20 mM Tris∙Cl buffer). The collected fractions (#), which were pooled after SDS-PAGE gel analysis are indicated. To increase purity of Mal d 1 in run 3, sub-fractions of small volume were collected and discarded.

The respective chromatograms, indicating the fractions are provided in Figure 2.

## 4. Expected Results

### 4.1. Quantity and Purity of the r-Mal d 1-His and r-Mal d 1 

**Figure 3:**
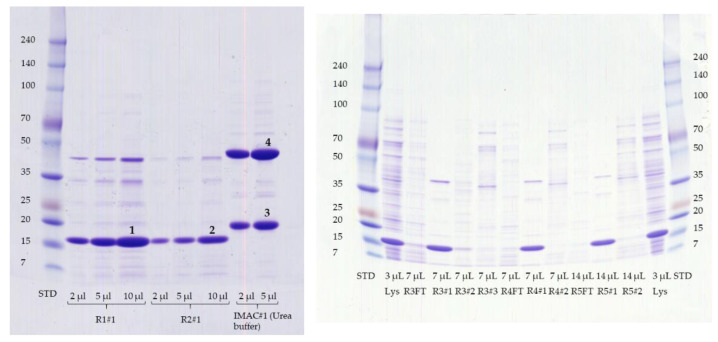
SDS-PAGE gels for pooled stored (−20 °C) fractions (#), lysate (Lys) and FT (flow-through) indicated in Figure 1 of run (R) 1 and 2 (left) and run 3 to 5 (right). Samples were diluted with water 1 to 5 prior to denaturation. The respective volume applied to the gel is specified in the figure. The numbers 1–4 indicate the bands used for in-gel digestion (see Section 4.2.1 and Appendix B.).

**Figure A1:**
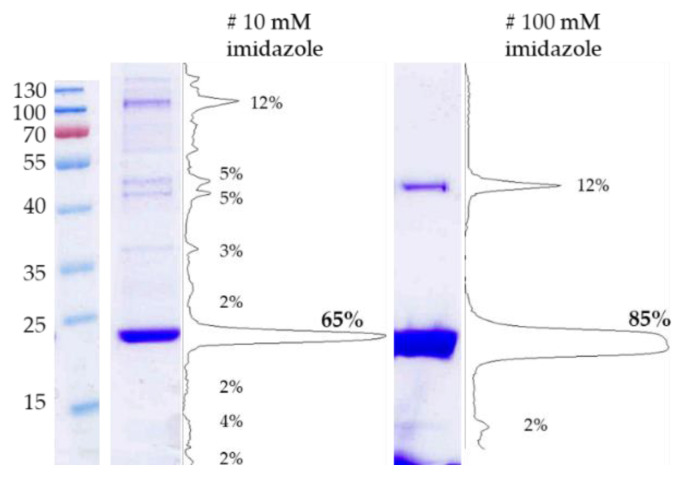
SDS-PAGE gel and determined purity for the pooled fractions eluted with Tris buffer containing 10 mM (**left**) and 100 mM imidazole (**right**).

Pooled fraction volumes and protein concentrations for IMAC and IEC purification are shown in Table 2. To evaluate the purity and to identify the r-Mal d 1, 10 µL of the pooled fractions was mixed with 40 µL millipore water and 12.5 µL SDS Laemmli sample buffer (fourfold concentrated), denaturized at 95 °C and separated on SDS-PAGE gel, which was stained with Coomassie Brilliant Blue. After washing in aqueous acidic isopropanol (80/10/10, *v*/*v*/*v*), the gels were scanned, and the purity was evaluated by Fiji [20]. If required, the bands were used for in-gel tryptic digestion (Figure 3). The fraction of the IMAC clean up eluted with 10 mM imidazole is of lower r-Mal d 1–His concentration (10.4 mg/mL) and less purity (65% r-Mal d 1–His) than the fraction with 100 mM imidazole (14.4 mg/mL and 85% r-Mal d1–His) (Table 2, Figure A1).

**Figure 4:**
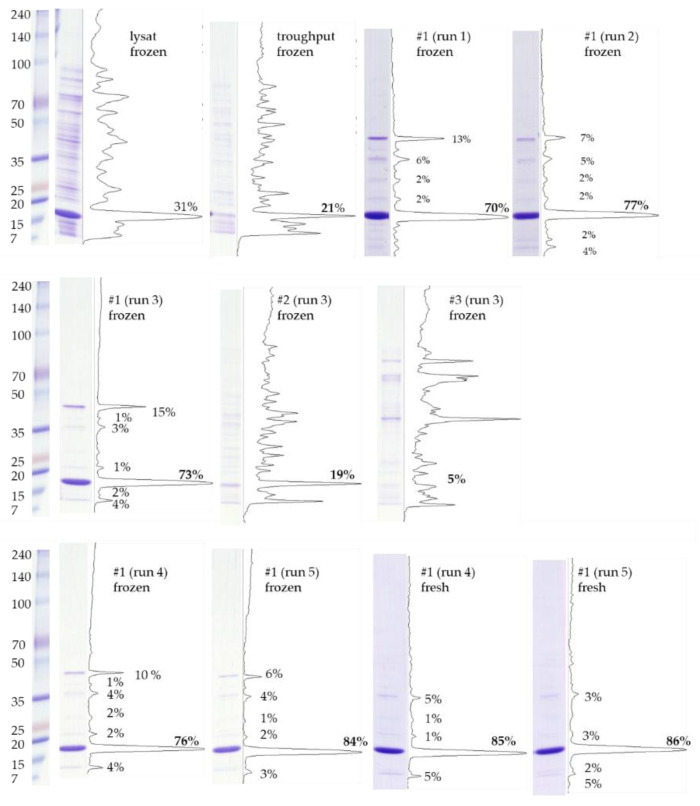
Purity of r-Mal d 1 in pooled fractions estimated from stained SDS-PAGE gel analysis (Figure 3) by Fiji. The cut out of #1 for run 4 and 5 is taken from the SDS-PAGE gel performed immediately after purification.

The r-Mal d 1 without an additional polyhistidine-tag was purified on a strong anion-exchange chromatography raisin. The high-resolution SuperQ-650S SkillPak^TM^ usually contains *High Capacity Ion Exchanger* with a particle size of 35 µm. The GigaCap Q-650M SkillPak^TM^ is filled with an *Ultra High Capacity Ion Exchanger* polymer-modified resin with a larger content of coupled ionic groups. Therefore, increased particle size is sufficient (75 µM), permitting higher flow rates. The first runs showed that TOYOPEARL^TM^ GigaCap Q-650M performed somewhat better (Figure 3, run 1 vs. run 2); therefore, the procedure was optimized exclusively for this column (Table 1). Using a stepwise gradient (run 4) slightly increased the volume of fraction containing r-Mal d 1 with a purity higher than 85% but mainly shortened the procedure to 40 min (Figure 2, Table 1). The r-Mal d 1 was concentrated in fraction #1 (Figure 3). However, negligible amounts of r-Mal d 1 were also found in the flow-through and fraction #2. The purity of the fractions was estimated by using an image-processing program (Fiji) [20]. The fractions used for SDS-PAGE gel analysis immediately after clean-up (run 4 and 5 #1 fresh) showed a Mal d 1 purity higher than 85% (Figure 4) with 3–4 impurities, below 5%. The SkillPak^TM^ column’s optimal loading volume is 5 mL lysate obtained from 1 g cells pellet. Lower amounts do not improve purity or yield. TOYOPEARL^TM^ GigaCap Q-650M are available in greater dimensions enabling possibilities for upscaling.

Avoiding dimerization during storage, the purity around 90% is sufficient for interaction studies (e.g., Isothermal Titration Calorimetry, Saturation Transfer Difference NMR experiments and SAR by NMR) if the impurity for each individual protein is below 5%. For immunological studies often a purity ≥95% is suggested. However, distinct values for the Mal d 1 purity are not specified in the references cited. Nevertheless, further purification of the low Mal d 1 amounts required for these investigations might be easily performed by an additional HPSEC step. 

### 4.2. Characterization 

#### 4.2.1. Verification of r-Mal d 1 and Characterization of an Impurity Formed during Storage by Mass Spectrometry after in Gel Digestion

**Figure 5:**
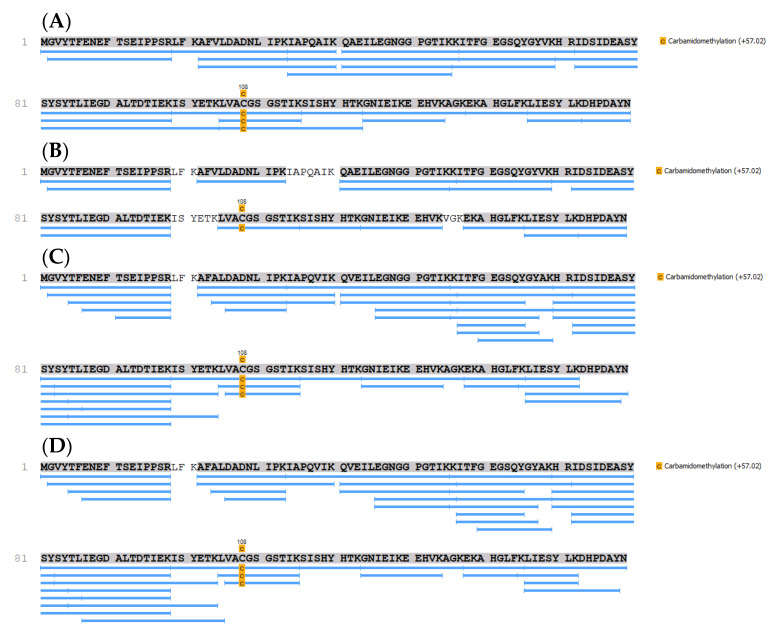
Sequence coverage determined by mass spectrometric of tryptic in-gel digests of bands excised from an SDS-PAGE (Figure 3 and Appendix C.). (**A**), band 1, run 1#1, stored at −20 °C; (**B**), band 2, run 2 #1, stored at −20 °C; (**C**) band 3 and (**D**) band 4, both belong to the IMAC fraction eluted with Tris buffer containing 10 mM imidazole, which was concentrated and stored in urea buffer at −20 °C.

Due to differences in the apparent molecular weight among the r-Mal d 1 and r-Mal d 1-His and the successive formation of a compound of higher molecular weight in-gel digestion followed by mass spectrometry is applied, to verify the identity of band 1 to 4 (Figure 3). The peptides generated by the tryptic digestion of band 1 and 2 as well as 3 and 4 indicate the expressed r-Mal d 1 as the primary protein (Figure 5). The sequence coverage is excellent for band 1 (r-Mal d 1, 100%, run 1#1), for band 3 (r-Mal d 1-His, 98%), and good for band 2 (88%, run 2#1). Based on the peptides found for band 4 (98%), it is identified as an aggregate of r-Mal d 1-His, formed after the purification step during buffer exchange/concentration and storage. The formation of dimers under physiological conditions has been already mentioned by Roulias et al. Like Bet v 1, this group showed by SDS-PAGE that Mal d 1 tends to form oligomers with a size of 47 kDa probably by disulfide-mediated aggregation. They determined a proportion of 34% dimers by High Pressure Size Exclusion Chromatography (HPSEC), which is comparable to our result. 

#### 4.2.2. Verification of the Correct Folding by Circular Dichroism Spectroscopy

**Figure 6:**
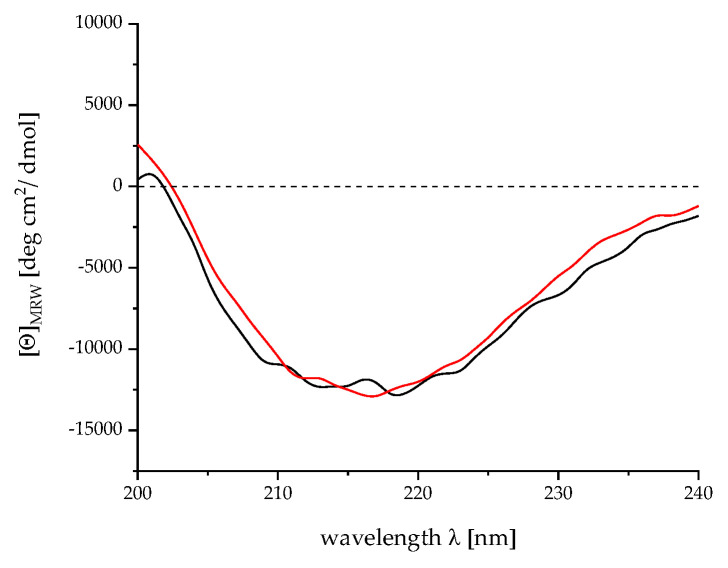
CD spectra for r-Mal d 1 (blue) *versus* r-Mal d1-His (orange).

The correct folding of r-Mal d 1 and r-Mal d 1–His is monitored by CD spectroscopy and secondary-structure elements are calculated (see Appendix D). CD spectra of r-Mal d 1 and r-Mal d 1–His are analogous (Figure 6). In accordance, their shape agrees with the spectra provided in the literature [9,18]. The analysis of the secondary structure results in 12%/19% α-helices, 39%/36% ß-sheet, 17%/19% turn and 32%/26% random coil for r-Mal d 1 and r-Mal d 1–His, respectively. These data conform to the secondary-structure information (25% α-helix, 35% ß-sheet) calculated from NMR spectra [2].

#### 4.2.3. Impact of Storage Conditions and Freeze-Thawing Process on Dimer Formation 

During storage at −20 °C and the freeze-thawing process, purity decreased due to the formation of a new compound with higher molecular weight (Figure 3 and Figure 4, run 3–5 #1, frozen). Despite an apparent molecular weight above 34 kDa, this compound was characterized by mass spectrometry (see Section 3.3.1.) as a r-Mal d 1 dimer. In addition, by adding 10 mM dithiothreitol (DTT) to the sample 10 min before denaturation, the band disappeared (Figure 6). With storage time, the dimer is probably formed by a disulfide bridge due to oxidation of the cysteine residues [18]. However, adding reducing agents during storage to prevent this dimerization is problematic because these substances might interfere in further experiments using the r-Mal d 1. 

**Figure 7:**
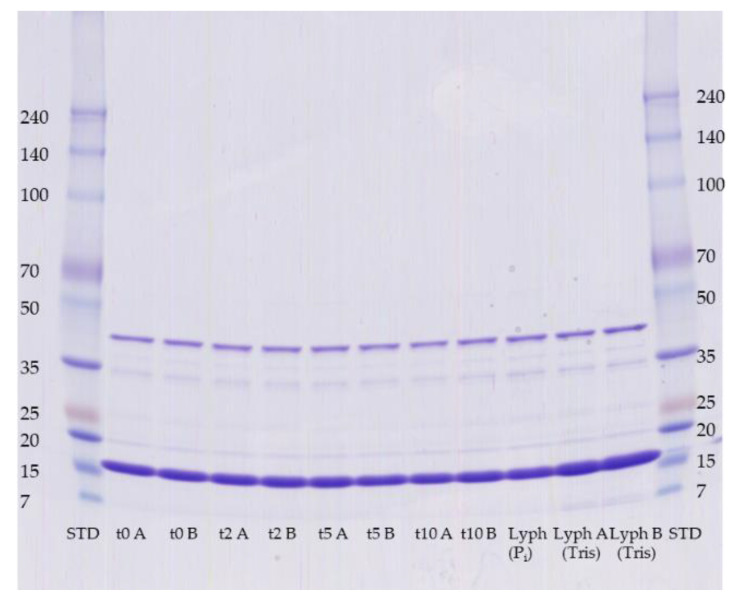
Impact of freeze-thawing and freeze-drying on the formation of the r-Mal d 1 dimer for independent samples (A, B). Freeze-drying was performed with r-Mal d 1 in Tris buffer containing the NaCl as a residue of the ion-exchange chromatography and after re-buffering in phosphate buffer. Samples were diluted with water 1 to 5 prior to denaturation, the volume applied to the gel was 14 µL.

To evaluate the impact of freeze and thawing effects, we simulated 10 cycles in a one-day experiment and observed dimer formation by SDS-PAGE gel analysis and CD spectroscopy. In addition, the process of freeze-drying of Mal d 1 in Tris buffer was studied (Figure 7). 

**Figure 8:**
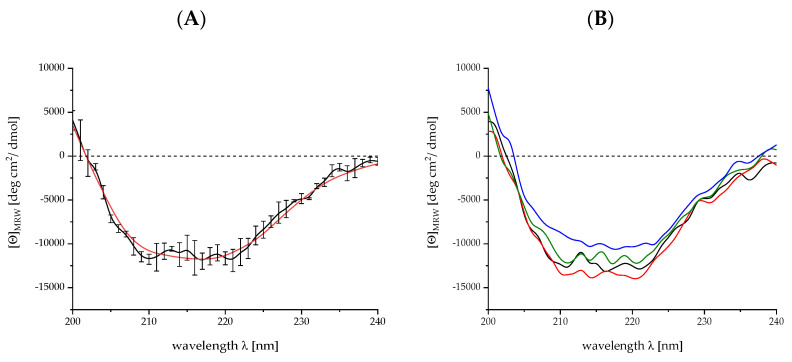
CD spectra for r-Mal d 1 for three independent measurements (**A**), data black, fit according to [21] in red) and at different cycles for freeze and thawing (**B**); 0, black; 2, green; 5, orange; 10, blue).

The impact of freeze-thawing (up to 10 times within one day) and freeze-drying was less relevant for the dimer formation with contents of 19 ± 1% and 72 ± 2% for the dimer and monomeric r-Mal d 1, respectively (Figure 6, purity evaluation not shown). In addition to the negligible impact of the freeze and thaw processes on dimer formation, no significant differences are observed in the respective CD spectra (Figure 8). The proportion of secondary-structure elements is constant within the standard deviations of independent measurements at the beginning (Table A1).

**Figure 9:**
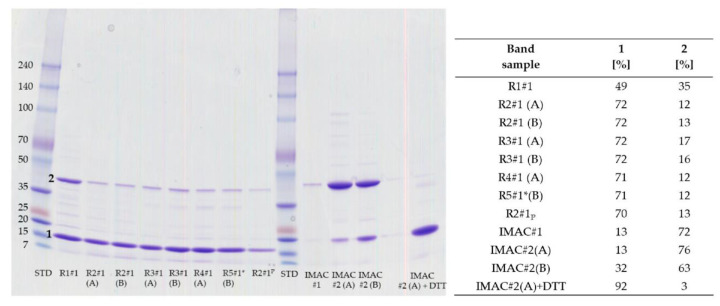
Impact of storage at −20 °C in Tris buffer containing 100mM NaCl and phosphate (Pi, R2#1) and urea buffer (IMAC#1 and #2) on the formation of the r-Mal d 1 and r-Mal d 1–His (IMAC; #1, 10 mM imidazole; #2 100 mM imidazole) dimer. * The sample volume used for the SDS-PAGE gel was doubled for run 5 #1. The relative proportion for the monomer (1) and dimer (2) are determined from stained gels by Fiji.

Dimer formation seems to increase with storage time (Figure 9) independently, whether in Tris or phosphate buffer. This effect was more pronounced for run 1 #1 (longest storage time) than for all other runs. Urea buffer was detrimental, forcing the formation of dimers during storage. However, the addition of 10 mM DTT (sample IMAC#2(A)+DTT, Figure 9) to the sample 10 min before denaturation leads to the monomer of high purity. The process of freeze-drying does not increase dimer formation. However, CD spectra indicate that folding is affected (data not shown).

## Figures and Tables

**Table 1:** Purification of r-Mal d 1 by ion-exchange chromatography.

Run	Lysate [mL]	Column	Gradient ^1^
1	5	TOYOPEARL^TM^ SuperQ-650S	linear, 1.5 mL/min, 0 to 100% B for 15 CVs
2	5	TOYOPEARL^TM^ GigaCap Q-650M	linear, 2 mL/min, 0 to 100% B for 20 CVs
3	5	TOYOPEARL^TM^ GigaCap Q-650M	linear, 2 mL/min, 0 to 100% B for 25 CVs
4	5	TOYOPEARL^TM^ GigaCap Q-650M	stepwise, 2 mL/min, 13 % B for 5 CV, 100% for 5 CVs
5	2.5	TOYOPEARL^TM^ GigaCap Q-650M	stepwise, 2 mL/min, 10 % B for 5 CV, 100% for 5 CVs

^1^ A, equilibration buffer 20 mM Tris∙Cl, pH 7.5; B, elution buffer 1 M NaCl in 20 mM Tris∙Cl, pH 7.5; CV, column volume 5 mL.

**Table 2:** Fractions of purified r-Mal d 1 obtained with different methods.

Run	Volume ^1^ [mL]	Concentration ^1^ [mg/mL]	Yield Mal d 1 ^2^ [mg/Clean Up Step]
IMAC#1 ^3^	12.5	10.4	130 ^5^
IMAC#2 ^4^	25	14.4	360 ^5^
1	5	2.9	14.5 ^6^
2	16	2.0	32.0 ^6^
3	14	3.0	42.0 ^6^
4	18	2.9 ^7^	52.2 ^6^
5	18	1.3 ^7^	23.4 ^8^

^1^ Protein volume and concentration of pooled fractions containing Mal d 1 with a purity higher than 85% determined by measuring absorption at 280 nm with ε 0.844 mg/mL [https://web.expasy.org/cgi-bin/protparam/protparam1?P43211@noft@] (Spectrostar nano, BMG Labtech, Ortenberg); ^2^ provided a Mal d 1 purity of 85%; ^3^ IMAC, fraction eluted with 10 mM imidazole; ^4^ IMAC, fraction eluted with 100 mM imidazole; ^5^ yield for 8 g cell pellet used in one clean up step, ^6^ yield for 1 g cell pellet used in one clean up step; ^7^ without dimerization purity is around 90%, ^8^ yield for 0.5 g cell pellet used in one clean up step.

## Data Availability

Data sharing is not applicable to this article.

## Appendix B. In-Gel Digestion

In-gel digestion was carried out according to the protocol by Shevchenko et al. [22], with some modifications. The gel was rinsed with water for 10 min, and bands 1 to 4 (Figure 3) were excised with a scalpel in pieces of 2 × 2 mm. The pieces were incubated with 500 µL acetonitrile for 10 min. The solvent was discharged, and reduction was performed at 56 °C for 30 min with 40 µL 10 mM DTT in 100 mM ammonium hydrogen carbonate buffer. After cooling to room temperature, 500 µL acetonitrile was added. After 10 min the solvent was removed and 40 µL 55 mM iodoacetamide in 100 mM ammonium hydrogen carbonate buffer was added for alkylation. The samples were incubated for 20 min in the dark, followed by the addition of 500 µL acetonitrile. The solvent was discharged, and the pieces were washed with 700 µL 200 mM ammonium hydrogen carbonate buffer/acetonitrile (70/30 *v*/*v*) at 37 °C for 15 min. This step was repeated until the pieces were decolorized. The pieces were dried using a vacuum concentrator (30 °C, 1500 rpm) for approx. 30 min. Digestion of the protein was performed with 100 µL of aqueous trypsin (2 µg/mL) at 37 °C for 15 min. The excess of tryptic solution was removed after 15 min. The gel was incubated overnight at 37 °C and then sonicated with 20 µL water for 15 min to extract the peptides. After centrifugation for 30 s at 10,000× *g*, the supernatant was mixed with 10 µL H_2_O/acetonitrile/formic acid (91/9/0.3 *v*/*v*/*v*), mimicking chromatographic starting condition and subjected to HPLC−MS/MS analysis.

## Appendix C. Mass Spectrometric Identification

The tryptic in gel digests were separated by reversed-phase liquid chromatography on a YMC-Triart C18 column using an M3 MicroLC system at 40 °C and a flow rate of 7 μL/min. After injection of 5 µL, the peptides were eluted with aqueous formic acid (A, H_2_O/ formic acid 99.9/0.1 *v*/*v*) and formic acid in acetonitrile (B, acetonitrile/ formic acid 99/0.1 *v*/*v*). Starting with 3%, B was raised to 8% in 2 min, increased to 30% from 2 to 68 min and to 40% from 68 to 73 min. Then B was increased to 80% in 3 min and maintained at 80% for 3 min until it was decreased to 3% in 1 min and reconditioning for 8 min. LC–MS analyses were carried out on a quadrupole TOF mass spectrometer (TripleTOF™ 6600, SCIEX). Acquisitions were performed using Analyst^®^ TF 1.7 software (SCIEX). Standard DDA (data dependent acquisition) runs were performed and the data analyzed with PEAKS X+ (https://www.bioinfor.com/peaks-studio) matching the peptides with the data for Mal d 1 found in UniProt [https://www.uniprot.org/] and the sequence for the modified plasmid insert.

## Appendix D. CD Spectroscopy

Circular dichroisms spectroscopy was carried out with a JASCO-J815 spectrophotometer using a quartz cuvette of 0.1 cm path length. Far UV (200 to 240 nm) spectra with approx. 0.1 mg/mL protein in 100 mM phosphate buffer pH 7.5, diluted with 200 mM KCl (1/3 *v*/*v*) were recorded at 20 °C with 1 nm band width, 1 s response time and 1 nm data pitch. A total of 40 consecutive scans were averaged, and the baseline as the average of blank samples was subtracted from the spectra. Data are presented as mean residue molar ellipticity (*Θ*), and secondary-structure elements are calculated based on Provenchur and Glöckner (1981) [21]. Molar ellipticity is calculated according equation 1 by the use of 9.91 µM protein concentration, 0.1 mm for path length and *n* = 159 and 165 for the r-Mal d1 and r-Mal d1-His, respectively.

(A1) $$\left[\Theta \right]\left(deg\;\cdot \;cm^{2}\;\cdot \;dmol^{-1}\right)=\;\frac{ellipticity\;\left(mdeg\right)\;\cdot \;10^{6}}{path\;length\;\left(mm\right)\;\cdot \;\left[protein\right]\;\left(\mu M\right)\;\cdot \;\left(n-1\right)}$$

**Table A1:** Variation of secondary structure elements of purified r-Mal d 1 for independent samples ^1^ and alterations during the freeze and thaw process ^2^.

Secondary Structure Element [%]	t_0_A	t_0_B	t_0_B ^3^	t_2_B	t_5_A	t_5_B	t_10_A	Average	SD
α-helix	17	17	18	20	19	17	17	17.9	1.1
β-sheet	37	37	36	35	36	37	38	36.6	0.9
turn	19	19	19	19	19	19	21	19.3	0.7
random	28	27	26	26	27	27	24	26.4	1.2

^1^ independent Mal d 1 dilutions A and B prior to the freeze and thaw process; ^2^ sample was frozen and subsequently thaw for 2, 5 and 10 times; ^3^ t_0_B repeated measurement after sample storage at 7 °C overnight.
